# Public health and climate change: How are local authorities preparing for the health impacts of our changing climate?

**DOI:** 10.1093/pubmed/fdz098

**Published:** 2019-12-11

**Authors:** Sarah C Woodhall, Owen Landeg, Sari Kovats

**Affiliations:** 1 Public Health and Wellbeing Division, South Gloucestershire Council, Bristol BS37 5AF, UK; 2 London School of Hygiene and Tropical Medicine, London WC1H 9SH, UK; 3 Extreme Events and Health Protection, Public Health England, London SE1 8UG, UK; 4 NIHR Health Protection Research Unit (NIHR HPRU) in Environmental Change and Health, London School of Hygiene and Tropical Medicine, London WC1H 9SH, UK; 5 Department of Public Health, Environments and Society, London School of Hygiene and Tropical Medicine, London WC1H 9SH, UK

**Keywords:** climate change adaption, extreme weather events, climate resilience, emergency preparedness, resilience and response, health system resilience

## Abstract

**Background:**

Local authorities have a crucial role in preparing for the impacts of climate change. However, the extent to which health impacts are being prioritized and acted on is not well understood.

**Methods:**

We investigated the role of public health in adapting to climate change through: (i) a content analysis of local authority climate change adaptation strategies in South West England and (ii) semi-structured telephone interviews with local authority public health consultants and sustainability officers and a regional Public Health England representative (*n* = 11).

**Results:**

Adaptation strategies/plans varied in existence and scope. Public health consultants did not have an explicit remit for climate change adaptation, although related action often aligned with public health’s emergency planning functions. Key barriers to health-related adaptation were financial constraints, lack of leadership and limited public and professional awareness about health impacts.

**Conclusions:**

Local authorities in South West England have differing approaches to tackling health impacts of climate change, and the prominence of public health arguments for adaptation varies. Improved public health intelligence, concise communications, targeted support, visible local and national leadership and clarity on economic costs and benefits of adaptation would be useful for local authorities in preparing for the health impacts of climate change.

## Background

Climate change has enormous implications for human health. This includes direct effects, including heat-related illness, deaths due to high/low temperatures and the physical and mental health impacts caused by flooding. Indirect effects are also expected arising from interactions between the environment and populations, for example, through disruption of food supply, economies and international relations.^1^^,^^2^ In the UK, health-related impacts of climate change include those arising from higher summer temperatures, more frequent flooding, poorer air and water quality and changes in incidence of food, water and vector-borne disease. Disruption of health and social care services is also expected, both in terms of increased need for emergency response and effects on day-to-day running of services.^3^^,^^4^

Locall authorities are crucial to the delivery of actions to prepare for the impacts of climate change (termed ‘adaptation’) given their democratic mandate and wide remit in local areas, including roles in planning, the built environment and—in England—public health.^5^ There is a legal requirement for national government to undertake a risk assessment relating to climate change and produce a response action plan (the National Adaptation Programme, NAP^5^) every 5 years.^6^ However, in local government, there is no statutory duty for climate change adaptation and there is concern about whether local authorities are equipped to take necessary actions. For example, in 2018, the Environmental Audit Committee expressed concern that essential heatwave adaptation measures are not being delivered and that previously available funding for adaptation had been withdrawn and resources were not being updated.^7^

In this study, we combined key informant interviews with content analysis of local authority climate change adaptation strategies to investigate how local authorities are preparing for the health-related impacts of climate change and to identify ways of strengthening the role of public health in responding to the challenge of climate change.

## Methods

### Content analysis

We reviewed publicly available climate change adaptation strategies or action plans for upper-tier local authorities in South West England. Documents were identified by entering search terms (‘climate change’, ‘strategy’ and ‘adaptation’) into Google and into search functions on local authority websites. Each document was coded under four pre-defined categories: (i) health impacts of climate change mentioned (e.g. heat deaths), (ii) risk assessment (e.g. mentions of risk scoring), (iii) planned health-related adaptation actions and (iv) information sources cited regarding health impacts/risk assessment.

### Semi-structured interviews

A convenience sample of public health consultants (PHCs) and sustainability officers (SOs) working in local authorities in South West England was recruited for interviews. Participants were invited by email via a regional network of local authority PHCs with lead responsibility for health protection (*n* = 13 local authorities) and a network of SOs in a sub-region of the South West (*n* = 4). Participants were a mix of individuals known and not known to the researcher before commencing the interviews. Five PHCs from four local authorities and five SOs from four local authorities participated. In some cases, both a PHC and an SO from the same local authority participated, resulting in participants from six local authorities in total (five of which had a published adaptation strategy). One regional Public Health England (PHE) representative was also invited and participated.

Semi-structured telephone interviews were conducted using a pre-defined and piloted topic guide,^8^ covering the participant’s roles and responsibilities in relation to adaptation and what tools and resources they had found useful or like to see in the future. Participants were asked how their local strategy (if they had one) had been developed and about facilitators and challenges in developing or implementing an adaptation strategy. Interviews lasted around 30 minutes and were recorded and transcribed. Analysis was carried out using the Framework Method.^9^ All interview transcripts were read and an initial set of codes developed, which were then applied to each transcript. Coding was iterative, with codes added as each transcript was coded where necessary. Groupings of codes were developed to reflect the interview topics (e.g. role of public health teams, facilitators/barriers to adaptation) and depending on sub-themes which emerged.

Microsoft Excel was used to manage data from the interviews and content analysis. Interviews and analyses were undertaken by one female researcher (S.C.W., PhD) as part of an MSc project conducted while undertaking specialty training in public health.

### Ethics

Ethics approval was granted from the London School of Hygiene and Tropical Medicine MSc ethics committee (Ref. 14755). Interview participants were given written information about the study before consenting to participate.

## Results

### Content analysis

Climate change adaptation strategies/action plans were identified for nine of the 13 upper-tier local authorities in the South West. Ten documents from nine local authorities were included (Table 1). Publication dates ranged from 2006 to 2018.

**Table 1:** Health impacts identified and adaptation actions included in climate change adaptation strategies/action plans (*n* = 10 plans from nine local authorities)

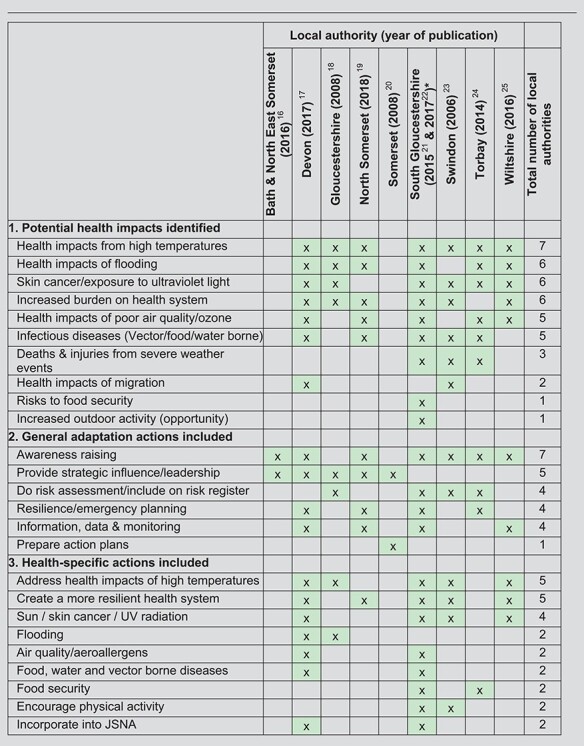

a In South Gloucestershire, a revised adaptation plan was in preparation at the time of data collection so both the previous plan and the draft revision were included.

``x'' denotes that health impact or adaptation action was found to be included in the adaptation/action plan.

The documents varied in the extent to which health impacts were included (Table 1, section 1). Two strategies/plans had no specific mention of health impacts, whereas others referred to a variety of impacts. Health impacts from high temperatures were most consistently included (7/9 local authorities), followed by health impacts of flooding, increased risk of skin cancer, and increased burden on the health system (all 6/9). In most cases, there was little detail provided about each health impact, and strategies did not quantify risks in terms of expected burden of disease in their local area. Most documents included little specific mention of evidence regarding health impacts of climate change. The most commonly cited document was the PHE publication, ‘Health Effects of Climate Change’.^10^

Actions specified as addressing a health risk varied by local authority (Table 1). Three documents included no health-specific actions, although two of these indicated that action plans would be prepared. The most commonly included general adaptation actions (Table 1, section 2) were to: raise awareness of climate change risks among the public/across the council (7/9) and provide strategic leadership (e.g. influencing cross-council discussions on business planning) (5/9). The most commonly included health-specific actions (Table 1, section 3) were: address health impacts of high temperatures (e.g. implementing the Heatwave Plan for England,^11^ provision of shaded areas and drinking water in urban areas, influencing planning decisions) (5/9) and create a more resilient health system (e.g. improved risk assessment, emergency planning, provision of information and procurement) (5/9).

### Semi-structured interviews

None of the PHCs interviewed had an explicit remit for health impacts of climate change, although several were involved in related work such as implementing the Heatwave Plan for England and emergency planning. Participants often emphasized the need for collaboration between departments, although the relationship between public health and SOs was often seen as one that could be strengthened. Several PHCs expressed uncertainty about who has responsibility for adaptation actions. In some cases, this lack of a clear remit had resulted in public health having a limited role in developing and implementing action plans from a health perspective.

‘I don't think climate change sits as an explicit issue [within public health] … I don't think there's any sort of championing of it by any senior officers or cabinet members from a health side.’ (P4, PHC)

This lack of a clear remit for public health is consistent with the experience from the PHE representative, who had rarely been consulted by local authority colleagues regarding adaptation. The perceived importance of public health arguments in adaptation also varied; some participants considered health impacts of climate change to be key to making the case for investment in adaptation whereas others saw public health arguments as secondary to expected impacts on infrastructure.

The links between climate change adaptation and emergency planning responses were considered key drivers for action in some instances. As well as having an existing framework through which risk assessment could be done and actions determined, emergency planning was considered noncontroversial. However, some saw the emergency planning role as limited as it focused on short-term logistics rather than taking a more preventive approach.

‘But now our emergency planning work is quite minimal. They do cover weather but its more [about] logistics … rather than thinking about why these events are happening and if there's anything we can do to reduce any of the influence of them in terms of investment.’ (P6, SO)

Some common facilitators and barriers to developing adaptation strategies and action plans emerged during the interviews. Almost all participants referred to insufficient resources and financial constraints as a key barrier to developing and implementing adaptation strategies. Participants felt that local authorities were often limited to providing only essential or mandated services.

‘… the greatest thing that local authorities … are concerned about at the moment is keeping the books balanced … that takes priority over everything else.’ (P1, PHC)

Another key barrier was the lack of both public discourse and professional awareness around the health impacts of climate change. As one participant put it,

‘the public health aspects of this are not yet in the public consciousness.’ (P10, SO)

Participants suggested that climate change lacked visibility within public health and felt that senior officers did not necessarily provide support for the issue or have a clear understanding of the local authorities’ responsibilities. Strong local and national leadership was considered crucial, for example, during discussions with developers or when advocating for adaptation interventions.

‘I think leadership both nationally, regionally and locally is really hugely important … when it's taken seriously [at a national level] both on the mitigation and the adaptation side, that does have a big impact.’ (P10, SO)

Several respondents talked about a change over the last decade both in public discourse and the political will to act on climate change. Participants perceived a reduction in recent years in central government’s focus on climate change and in staffing assigned to climate change-related work. The 2008 financial crisis, a change in national government in 2010 and reductions in local authority funding were cited as important reasons for the perceived shifts. Participants also referred to a reduction in support and guidance provided by national bodies.

‘there was an enormous shift under the coalition government … up until 2010 I could sit in meetings … and get the sense that this was a priority that number 10 took very seriously … That changed overnight in 2010 … it's a much much harder sell now.’ (P10, SO)

Having a national mandate to act was considered an important potential facilitator. For example, the change in reporting requirements through the loss of national indicators on climate change in 2010 (which were part of the local government National Performance Framework) was specifically mentioned by one participant when discussing reductions in climate change activity in the council.

The nature of climate change and adaptation was also seen as an inherent challenge to developing meaningful adaptation actions. Several participants talked about the mismatch of timescales between climate change impacts and local government planning and commissioning schedules and difficulties in measuring the impact of interventions or inaction.

‘There's something about the … mental discounting … about how far some of it is away - rather than more immediate concerns it gets supplanted [by] other things that you feel you need to also plan for.’ (P2, PHC)

‘We've got this enormous gap between the impact and the intervention … the two things are not directly related. We can't do something now and then see the impact of it directly tomorrow.’ (P10, SO)

Several participants talked about how climate change has been communicated and the importance of framing the issue to get sufficient traction for action. Participants often referred to climate-related events being presented as one-off weather events rather than being discussed as relating to climate change. This was seen as a missed opportunity by some. When talking about the potential for food shortages following the 2018 heatwave in England (which was ongoing at the time of the interviews), one participant reflected that:

‘Unless somebody gets out there and gets on the front foot and articulates it or one of our politicians grabs it … it will be number one about logistics … rather than about 'woah, hang on this is climate change, we've got to do something about it'. The wrong stories are being told.’ (P6, SO)

However, others felt that badging adaptation actions under the banner of climate change could be unhelpful as climate change was considered a controversial topic and one where the impacts are perceived as being far into the future.

Box 1 shows suggestions made by participants about what would help them to develop work on health-related climate change adaptation. Of note, several participants talked about the need for cost-benefit analyses to help them make the case for investment in climate change adaptation and the need for surveillance of health outcomes relating to climate change as well as monitoring of progress on adaptation actions. Participants also talked about the need for collaboration with other councils across relevant networks to facilitate shared learning and make the most of available resources.

## Discussion

### Main findings of this study

We found the role of public health within the field of climate change adaptation to vary across local authorities in South West England. PHCs within local authorities did not have an explicit remit for climate change adaptation, and health-related risks of climate change were not necessarily well-realized within local government. Local authority adaptation strategies and plans varied in existence and scope. There was minimal use of health-related evidence within the strategies/plans identified and the extent to which strategies referred to health impacts or incorporated health-related actions differed.

Box 1: Suggested resources or actions to support climate change adaptation

Financial implications

- Cost-benefit analysis
- Return on investment
- Update of Stern Review

Nationally collated guidance

- Synthesized evidence summaries
- Toolkits (e.g. similar to PHE suicide prevention toolkit)
- Guidance to support planning responses
- Checklists for incorporating into standard processes (e.g. commissioning)

Sector-led improvement/sharing good practice

- Sharing of policy wording
- Peer support
- Good practice examples of Joint Strategic Needs Assessments
- Good practice models of engagement with relevant organizations

Awareness-raising activities

- Highlighting public health aspects of climate change needing action now
- Webinars
- One-page summary of impacts, costs and trends

Surveillance/indicators

- PHE fingertips tool/climate change health impact profile
- Climate/weather impact monitoring system
- Annual report on impact of climate change
- Local impact on hospital admissions and healthcare usage

Adaptation actions and responsibilities often related to the emergency planning function within the council, which was seen to have an important role in risk management and to offer a means of addressing climate risks without controversy. However, the emergency planning approach was considered lacking in some respects as it does not generally take a preventive approach.

Interview participants identified financial constraints, a lack of strategic and political leadership, an absence of public and professional awareness about health impacts of climate change and challenges of demonstrating impact of interventions as key barriers to developing adaptation actions. Effective communication was considered crucial. Participants talked about the need for additional resources, support and leadership in relation to adaptation for health impacts of climate change. Increased collaborative working and sector-led improvement activities were seen as ways to improve work in this area, especially in the current financial climate.

### What is already known on this topic

There have been few previous studies to explore how local authorities are responding to climate change and health. A 2015 survey of 90 local authorities found that 62% of areas surveyed had an adaption plan in place.^12^ In their 2012–13 study, Porter et al. interviewed local authority environmental officers and found that staff felt equipped with sufficient information relating to adaptation but had limited capacity due to budget cuts and reduced staffing.^13^ Negev and Kovats explored perception of climate change adaptation specifically from a public health perspective through interviews with senior public health leaders in local authorities and PHE.^14^ They found that climate change was considered an important public health risk, but there was a gap between national research and local needs, with participants emphasizing the need for effective policy actions over an improved understanding of health impacts. Participants also identified several barriers to advancing public health adaptation actions, including low risk perception, a lack of political will and the short-term nature of political decision-making.

Our finding that emergency planning approaches were considered lacking in terms of prevention is consistent with a previous evaluation of the Cold Weather Plan for England.^15^ In that study, interview participants felt that the plan would be better led by public health rather than emergency planning to maintain emphasis on prevention of morbidity and mortality and long-term planning and to facilitate integration of actions across different departments. This is especially relevant given that the majority of public health impacts occur outside of the ‘emergency response’ window.

### What this study adds

By combining an assessment of published strategies with interviews from council officers and a PHE representative, this study provides insight into the current working practices, perspectives and challenges faced by those working in or with local authorities. It provides evidence about what people working in the fields of both health and sustainability would find useful when developing work in this area (Box 1) and allows us to make recommendations for future practice (Box 2).

Box 2: Recommendations and questions for future research

For national government (i.e. Public Health England, the Department for Environment, Food and Rural Affairs):

- Publish climate change-related health impact surveillance/indicators and regular reports on progress on adaptation.
- Produce climate change adaptation communications for local authority audiences, including evidence summaries, briefings and webinars.
- Facilitate sector-led improvement activities, including joint workshops between PHCs/Officers and SOs.
- Work with local authorities and relevant organizations to produce guidance on health needs assessment for climate change.
- Encourage visible, senior and national leadership to strengthen the role of public health and other health professionals in addressing climate change.

For local authorities:

- Consider including health impacts of climate change within Joint Strategic Needs Assessments.
- Incorporate health impacts of climate change into local emergency planning risk assessments.
- Identify opportunities for collaboration between Public Health, Sustainability/Environmental and Planning departments.

Future research questions:

- What are the most effective ways of communicating about climate change within local authorities and public health departments?
- What are the costs and benefits of health-related adaptation activities?

Our findings are generally consistent with the previous studies by Porter et al.^13^ and Negev and Kovats,^14^ although there were some points of departure. In contrast with Porter et al., there was evidence from our interviews of a knowledge gap among PHCs and SOs. Several participants called for evidence summaries to develop their own knowledge and influence others. Negev and Kovats found that participants were ‘mostly satisfied with the prioritization of climate change in PHE agenda’.^14^ In our study, participants were not asked directly about their views on PHE’s prioritization of climate change. However, beyond referencing PHE’s 2012 summary of Health Effects of Climate Change^10^ and the Heatwave Plan for England, there was little mention of leadership provided by PHE and a couple of participants specifically stated that PHE could provide more support in this area.

In the present study, several interview participants discussed the potential benefit of monitoring and reporting on adaptation indicators. In contrast to this position, the 2018 NAP states that local government’s existing reporting duties regarding flood-risk management, planning, emergency planning, and biodiversity ‘negates the need for further adaptation reporting’.^5^ Our findings challenge this assertion as they show a desire from the system to use reporting and public health intelligence to inform local decision-making.

### Limitations of this study

Given the approach to sampling, data collection and the relatively small number of participants involved, findings of the interviews are not generalizable to other councils or regions. However, the themes identified, combined with findings from the content analysis, provide useful insight into the current state of adaptation strategies from a public health perspective. Additionally, analysis was undertaken by a single interviewer who also coded all transcripts. It is feasible that having a second coder may have affected the themes identified, but this was not feasible within the resource constraints of the project, and a systematic approach to coding was taken to minimize coder bias.

The content analysis was limited to published climate change strategies or action plans from upper-tier local authorities, which were identified via a search engine or individual council websites. It is feasible that more extensive actions and evidence are included in other council instruments (e.g. local plans, standalone heatwave plans) or unpublished documents. Adaptation actions were only included where explicit reference to a health risk was made. Thus some actions that would have a potential impact on health were not included in the analysis. This is of particular importance for flooding-related risks. Several local authorities included plans for reducing the potential impact of flooding through changes to the built environment, but these were rarely referenced as addressing health risks per se in the general strategies. This study did not examine implementation, meaning it is not possible to appraise the impact of any specified action.

## Conclusion

Local authorities in South West England take a variety of approaches in tackling health impacts of climate change, although many of the challenges faced in developing work on adaptation were similar across different areas. Participants identified a range of resources, approaches and research that would support their work in this area. These include: improved public health intelligence; concise communications; targeted funding and support for local authorities; greater strategic and political leadership and a clear understanding of economic costs and benefits of adaptation measures. Such resources and measures could support long-term prevention and emergency planning in local authorities, enabling public health to advocate action on climate change adaptation.

## Funding

This work was supported by the National Institute for Health Research Health Protection Research Unit (NIHR HPRU) in Environmental Change and Health at the London School of Hygiene and Tropical Medicine in partnership with Public Health England (PHE) and in collaboration with the University of Exeter, University College London and the Met Office. S.C.W. is employed by Gloucestershire Hospitals NHS Foundation Trust, who provided financial support for her MSc. The views expressed are those of the authors and not those of the NHS, the NIHR, the Department of Health and Social Care or Public Health England.

## Notes

**Sarah C. Woodhall**, Specialty Registrar in Public Health

**Sari Kovats**, Associate Professor and Director

**Owen Landeg**, Principal Environmental Public Health Scientist
